# Differences in Treatment Response in Bronchial Epithelial Cells from Idiopathic Pulmonary Fibrosis (IPF) Patients: A First Step towards Personalized Medicine?

**DOI:** 10.3390/antiox12020443

**Published:** 2023-02-10

**Authors:** C. Veith, M. A. Schneider, L. Maas, A. van der Vliet, F. J. van Schooten, M. Kreuter, M. Meister, A. W. Boots, N. Kahn

**Affiliations:** 1 Department of Pharmacology and Toxicology, NUTRIM School of Nutrition, Translational Research and Metabolism, Faculty Health, Medicine and Life Sciences, University of Maastricht, P.O. Box 616, 6200 MD Maastricht, The Netherlands; 2 Translational Lung Research Unit, Thoraxklinik, Heidelberg University Hospital, 69126 Heidelberg, Germany; 3 Translational Lung Research Center Heidelberg, 69120 Heidelberg, Germany; 4 Department of Pathology & Laboratory Medicine, Larner College of Medicine, University of Vermont, Burlington, VT 05405, USA; 5 Center for Interstitial and Rare Lung Diseases, Pulmonary and Respiratory Critical Care Medicine, Thoraxklinik, Heidelberg University Hospital, 69120 Heidelberg, Germany

**Keywords:** IPF, primary bronchial epithelial cells, nintedanib, pirfenidone, saracatinib, personalized medicine

## Abstract

Idiopathic pulmonary fibrosis (IPF) has a detrimental prognosis despite antifibrotic therapies to which individual responses vary. IPF pathology is associated with oxidative stress, inflammation and increased activation of SRC family kinases (SFK). This pilot study evaluates individual responses to pirfenidone, nintedanib and SFK inhibitor saracatinib, markers of redox homeostasis, fibrosis and inflammation, in IPF-derived human bronchial epithelial (HBE) cells. Differentiated HBE cells from patients with and without IPF were analyzed for potential alterations in redox and profibrotic genes and pro-inflammatory cytokine secretion. Additionally, the effects of pirfenidone, nintedanib and saracatinib on these markers were determined. HBE cells were differentiated into a bronchial epithelium containing ciliated epithelial, basal, goblet and club cells. NOX4 expression was increased in IPF-derived HBE cells but differed on an individual level. In patients with higher NOX4 expression, pirfenidone induced antioxidant gene expression. All drugs significantly decreased NOX4 expression. IL-6 (*p* = 0.09) and IL-8 secretion (*p* = 0.014) were increased in IPF-derived HBE cells and significantly reduced by saracatinib. Finally, saracatinib significantly decreased TGF-β gene expression. Our results indicate that treatment responsiveness varies between IPF patients in relation to their oxidative and inflammatory status. Interestingly, saracatinib tends to be more effective in IPF than standard antifibrotic drugs.

## 1. Introduction

Idiopathic pulmonary fibrosis (IPF) is a progressive and irreversible lung disease of unknown etiology, characterized by scarring of the lung tissue associated with a high burden of disease and a detrimental survival rate [1,2] In addition to exposure to environmental factors including cigarette smoke and asbestos, other factors such as genetic predisposition or premature aging are risk factors for IPF [3]. The pathogenic mechanisms underlying IPF are still mainly unclear, but the current paradigm is that a disrupted homeostasis of epithelial cells caused by damage from various triggers [3] plays an important role in the development of the disease. Recurrent epithelial injury leads to aberrant wound-healing responses and eventually causes the remodeling of the lung structure by promoting epithelial cell apoptosis and excessive collagen deposition in the alveolar space [4]. Furthermore, IPF is associated with an increased oxidant burden and diminished expression of antioxidant enzymes, including heme oxygenase-1 (HO-1), superoxide dismutase (SOD), catalase (CAT), glutaredoxin and glutamate cysteine synthase (γGCS, the rate-limiting enzyme in glutathione synthesis) [5,6], which is thought to further enhance epithelial injury. An important source of reactive oxygen species (ROS) in the lungs is the family of NADPH oxidases (NOXes). The family member NOX4 has been indicated to be upregulated in the lungs of IPF patients [7] in both epithelial cells and (myo)fibroblasts and is thought to promote alveolar epithelial cell (AEC) death, (myo)fibroblast differentiation and collagen deposition [8].

Recently, two clinically approved antifibrotic drugs, pirfenidone and nintedanib, were shown to ameliorate the course of the disease by slowing down its progression of [9], although they are minimally effective in curing or reversing disease progression. Pirfenidone is suggested to have antioxidative, anti-inflammatory and antifibrotic effects, but its exact working mechanisms and targets remain to be elucidated [9,10,11,12]. Nintedanib, originally developed as an antineoplastic / antiangiogenic drug, intracellularly blocks receptor tyrosine kinases, including the platelet-derived growth factor receptor (PDGFR), the fibroblast growth factor receptor (FGFR) and the vascular growth factor receptor (VEGFR)-2, by binding to their intracellular ATP binding pocket [9]. Nintedanib possesses antifibrotic effects as it inhibits the actions of fibroblasts [13], but its exact working mechanisms also still remain to be elucidated. Additionally, the effect of both of these drugs on the lung epithelium is not clear yet, as most studies thus far have investigated their influences on (myo)fibroblasts [11,14]. Recently, a study has demonstrated that low concentrations of pirfenidone (250 µM) and nintedanib (1 µM) also reduce fibrotic gene expression in isolated murine alveolar epithelial cells [15]. This limited knowledge regarding their mode of action, combined with the fact that IPF is a very heterogeneous disease, makes it very difficult to predict the effects of either pirfenidone or nintedanib in individual patients.

Recently, the non-receptor tyrosine inhibitor saracatinib (AZD0530, AstraZeneca), which inhibits SRC family kinases (SFK) and BCR-ABL kinases, has been suggested as a potential treatment strategy for IPF [16,17,18]. SFK are involved in various signaling pathways important for cellular homeostasis, including cell differentiation and proliferation [19], pathways that are often dysregulated in lung fibrosis [20]. It has been shown that SFK are activated in response to TGF-β and that inhibiting SRC kinases using AZD0530 in an experimental model of pulmonary fibrosis reduced the fibrotic area as well as collagen deposition in the lung [20], and might even be more efficacious than pirfenidone or nintedanib (Anghari et al., 2022). Based on these recent observations, a clinical trial investigating the possible beneficial health effects of saracatinib in IPF is currently ongoing (https://clinicaltrials.gov/ct2/show/NCT04598919), accessed on 11 November 2022.

IPF pathology is now commonly considered an epithelial-driven and fibroblast-activated process with mild inflammation [21], and effective treatment of IPF will involve targeting of not only fibroblasts but also aberrant epithelial cells that are present in fibrotic regions [22]. Interestingly, saracatinib treatment has recently been shown to modify profibrotic changes in airway basal cells, indicating a possible role for enhanced SFK activation in IPF epithelium [18,23]. Hence, it is worthwhile investigating the effects of antifibrotic drugs, including the potential antifibrotic saracatinib, on lung epithelium cells as well. This pilot study was designed to evaluate the antioxidative and anti-inflammatory effects of SFK inhibitor AZD0530 in differentiated bronchial epithelial cells (HBE) from IPF patients in comparison with those of pirfenidone and nintedanib. To this end, we examined primary HBE cells isolated from non-treated IPF patients as well as non-IPF patients and evaluated the effects of treatment with pirfenidone, nintedanib or saracatinib on the expression of NOX4 and profibrotic genes, as well as other genes involved in redox homeostasis and pro-inflammatory cytokine production.

## 2. Materials and Methods

### 2.1. Patient Characteristics

Patients were recruited at the Center for Interstitial and Rare Lung Diseases, Thoraxklinik, University of Heidelberg (see Table 1 for their characteristics). All patients gave written informed consent, and the study was approved by the ethics committee of the University of Heidelberg, Germany (vote S-538/2012).

Four patients diagnosed with IPF according to the ATS/ERS consensus criteria [24] were included in the study. Two were ex-smokers, one never smoked and one was an active smoker. At the time of sampling, none of the patient has received treatment yet. For the control non-IPF group, 3 patients (2 males, 1 female) undergoing bronchoscopy for further investigation of indeterminate pulmonary nodules were included (Table 1). ELF was obtained from a noninvolved segment from the contralateral lung, opposite the solitary lesion, to minimize potential influences of the suspected malignancy. In the control group, two patients were smokers and one was an ex-smoker.

### 2.2. Collection and Culture of Bronchial Epithelial Cells

Human bronchial epithelial (HBE) cells were obtained from endobronchial lining fluid (ELF) of IPF patients and controls by minimally invasive bronchoscopic microsampling (BMS) during bronchoscopy from subsegmental airways as previously described [25] After BMS, primary HBE cells were centrifuged for 5 min at room temperature at 300× *g* to elute sampled cells from sponges. Afterwards, cells were resuspended and expanded in DMEM/F12 media (Gibco, Carlsbad, CA, USA) supplemented with bovine pituitary extract (4 µL/mL), epidermal growth factor (10 ng/mL), insulin (5 µg/mL), hydrocortisone (0.5 µg/mL), triiodo-L-thyronine (6.7 ng/mL) and transferrin (10 µg/mL) (PromoCell, Heidelberg, Germany), sodium selenite (30 nM, Sigma, St Louis, MO, USA), ethanolamine (10 µM, Sigma), phosphorylethanolamine (10 µM, Sigma), sodium pyruvate (0.5 µM, Gibco), adenine (0.18 mM, Sigma), Hepes (15 mM, Gibco, NY, USA), 1x GlutaMAX (Gibco), and 10 µM Rock-inhibitor (StemCell, Cologne, Germany), as described previously [26].

**Figure 1:**
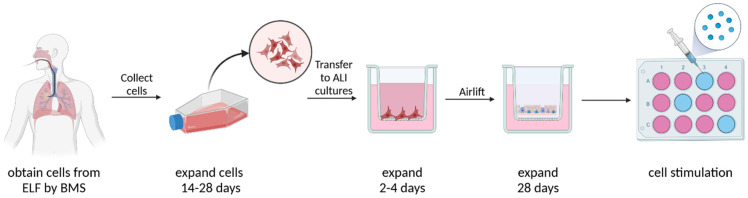
Culture method of HBE obtained by BMS. HBE cells were obtained from ELF by BMS and afterwards expanded in submerged cultures. After approximately 14–28 days, cells were transferred to tissue culture inserts for ALI culture. After reaching confluency after 2–4 days, cells were airlifted and maintained at ALI until a pseudostratified epithelium had developed. At day 28, cells were stimulated with pirfenidone, nintedanib or saracatinib. Finally, RNA, proteins and supernatants were collected. Created using BioRender.com.

After reaching confluency, cells were transferred to 12-well tissue culture inserts and plated at 90.000 cells/insert (ThinCert™, 0.4 µm pores, Greiner BioOne, Frickenhausen). After 2–3 days, cells were subjected to air–liquid interface (ALI) culture by removing the medium from the apical chamber and adding PneumaCult-ALI media (StemCell, Cologne, Germany) to the basal chamber only. Differentiation into a pseudostratified mucociliary epithelium was achieved after approximately 24–27 days. At day 28, cells were stimulated for 24 h with different concentrations of pirfenidone (1 mM, 500 µM, 100 µM), nintedanib (1 µM, 0.1 µM, 0.01 µM) or saracatinib (10 µM, 1 µM, 0.1 µM) (all Selleck Chemicals, Houston, TX, USA) that were added to the basolateral side of the medium to mimic the systemic administration of these drugs to patients. After this incubation, RNA, proteins and supernatants were collected (see Figure 1).

### 2.3. Confocal Imaging

To validate differentiation to a pseudostratified mucociliary epithelium, ALI cultures were stained with specific antibodies. The cells were washed with PBS and fixed on slides with 4% paraformaldehyde for 20 min at 4 °C. Afterwards, mounted slides were rinsed with PBS and incubated in PBS containing 0.2% Triton X-100 for 2 min. Slides were then cut in 4 pieces and washed with PBS, blocked in PBS containing 1% bovine serum albumin (BSA) for 30 min, and stained with antibodies against keratin 5 (1:50, #HPA059479, Sigma-Aldrich, St. Louis, MI, USA), tubulin-β4 (1:100, #T7941, Sigma-Aldrich, St Louis, MO, USA), CC10 (1:300, #RD181022220-01, BioVendor, Heidelberg, Germany) and mucin 5AC (1:300, Abcam, #ab3649, Berlin, Germany) overnight at 4 °C. The next day, slides were washed with PBS and blocked for 10 min in PBS/1% BSA. Alexa Fluor secondary antibodies 488 and 594 (1:300, Invitrogen, Carlsbad, CA, USA) were added, and slides were incubated for 45 min at 37 °C, followed by Hoechst staining (1:10,000, #H1399, Invitrogen, Waltham, MA, USA) for 10 min at room temperature. Images were obtained with confocal microscopy (Leica TCS SP5) and Leica Application Suite X software.

### 2.4. ELISA

Secretion of interleukin (IL)-8 and IL-6 in cell culture supernatants was determined using human ELISA DuoSet kits (R&D Systems, Minneapolis, MN, USA) according to the manufacturer’s instructions.

### 2.5. MTT Assay

Cells were plated in a 96-well plate, grown to 70% confluency and afterwards stimulated for 24 h. The next day, MTT (Sigma-Aldrich) solution (final concentrations 0.5 mg/mL) was added to all wells and incubated for 1 h at 37 °C in the dark. Cells were washed with HBSS, and DMSO was added to solubilize the formed formazan. Afterwards, the plate was incubated for 30 min on a shaker, and absorbance was measured at λ = 540 nm.

### 2.6. LDH Assay

LDH cell death assay was used according to the instructions of the manufacturer (Thermo Scientific, Waltham, MA, USA).

### 2.7. RNA Isolation and RT-PCR

RNA was isolated and purified using the RNeasy mini kit (Qiagen, Venlo, The Netherlands) according to the manufacturer’s instructions. The RNA concentration was determined using a NanoDrop spectrophotometer (Thermo Scientific), after which cDNA was synthesized from 500 ng isolated RNA using IScript (Biorad, Hercules, CA, USA) according to the manufacturer’s instructions. RT-PCR was performed using SYBR Green PCR Supermix (BioRad) with 4.4 μL of 50 times diluted cDNA and 0.5 μmol/L predesigned primers. PCR amplifications were carried out for up to 55 cycles of denaturation (95 °C, 10 s) and annealing/elongation (60 °C, 60 s) for selected genes (Table 2). The gene expression was normalized to the housekeeping gene β-actin and quantified according to the 2^−∆∆Ct^ method to relatively quantify the expression of genes of interest. These genes included the ROS-producing enzyme NOX4 as well as selected antioxidant genes (Table 2).

### 2.8. Statistical Analysis

All quantitative data were represented as means ± SEM. Statistical differences between groups were evaluated by means of 1-ANOVA analysis corrected with Bonferroni’s post hoc analysis or by means of Student’s t-test in GraphPad Prism software (version 7.3; GraphPad Software, La Jolla, CA, USA) and considered significant at a *p* value less than 0.05.

## 3. Results

**Figure 2:**
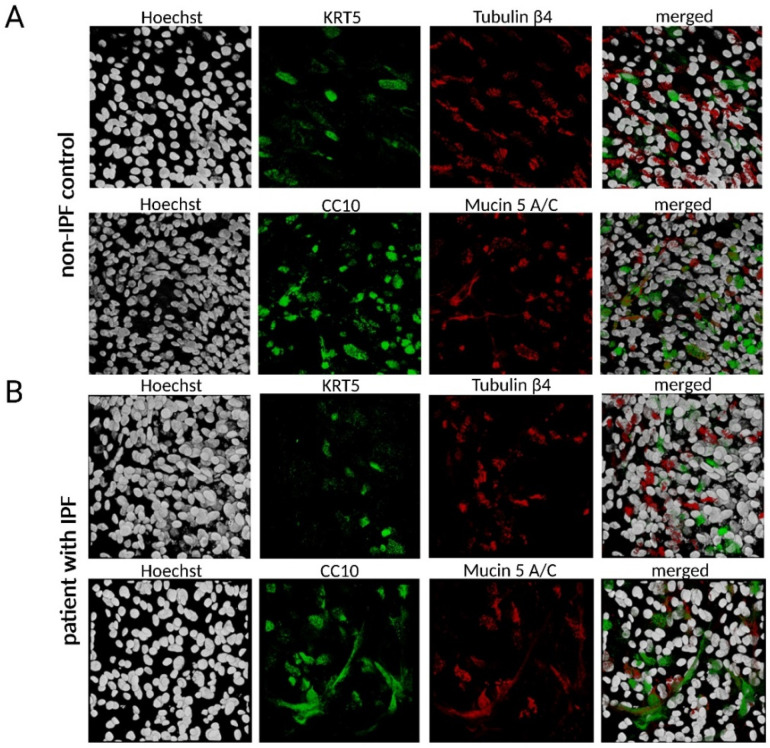
Differentiated HBE cells. (**A**) Top view of BMS-derived ALI cell cultures stained with Keratin 5 (green, basal cells) and Tubulin-β4 (red, ciliated cells) as well as CC10 (green, club cells) and Mucin 5A/C (red, goblet cells) from non-IPF controls (**A**) and IPF patients (**B**). Scale bar = 100 µm.

Human bronchial epithelial (HBE) cells were isolated from the endobronchial lining fluid (ELF) of patients with IPF and non-IPF controls by BMS and grown in ALI cultures. After 28 days of ALI, a complete bronchial airway epithelium consisting of ciliated epithelial, goblet, club and basal cells has developed. The HBE cells were stained with specific antibodies and confocal microscopy was performed showing the successful differentiation of BMS-derived cultures (Figure 2) from controls and patients with IPF. Keratin 5 staining, indicating basal cells, appeared mostly at the bottom of the cultures, whereas tubulin-β4 staining, indicating ciliated epithelial cells, was mostly distributed at the apical surface of the cultures. CC10 (club cells) staining and mucin 5A/C staining were evenly distributed throughout the culture. Although airway basal cells from IPF patients may differ from those of healthy subjects [18], no major differences were observed between ALI cultures from BMS-obtained cells from IPF patients and those from non-IPF controls (Figure 2).

**Figure 3:**
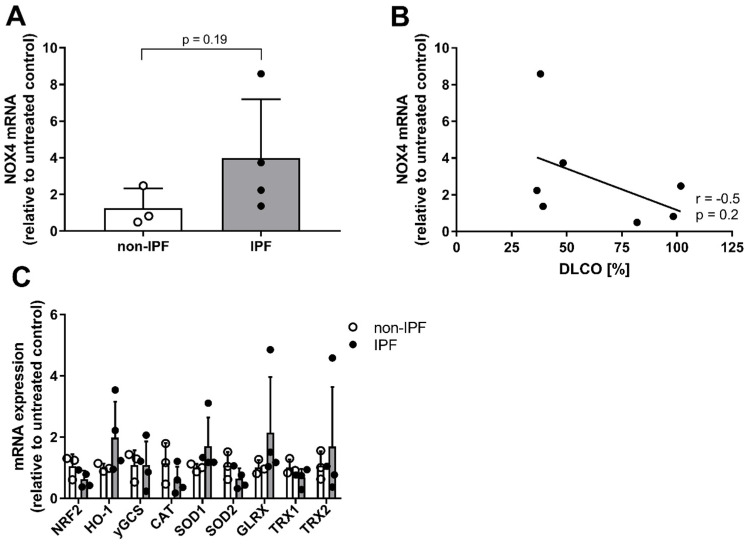
HBE cells derived from IPF patients have different gene expression levels of NOX4 and endogenous antioxidants. (**A**) NOX4 expression levels in HBE cells from IPF patients (*n* = 4) and controls (*n* = 3). Data are presented as mean ± SD. (**B**) Correlation of NOX4 expression with DLCO (%). (**C**) Antioxidant gene expression in HBE cells from IPF patients (*n* = 4) and controls (*n* = 3). Data are presented as mean ± SD. Gene expression is expressed relative to non-IPF controls.

Previous studies have indicated that epithelial cells from patients with IPF have an increased expression of the NADPH oxidase NOX4 that enhances epithelial oxidant production [27], whereas certain antioxidant systems are decreased [5,28,29,30,31,32], thereby causing a redox imbalance. Therefore, we assessed mRNA expression of NOX4 as well as various antioxidant genes in HBE cells from both controls and IPF patients. NOX4 was indeed upregulated in epithelial cells derived from patients with IPF in comparison to control cells (Figure 3A), but its expression differed notably between individual IPF patients (*p* = 0.19). Additionally, NOX4 mRNA expression was negatively correlated with the diffusing capacity for carbon monoxide (DLCO) (Figure 3B), although this was not statistically significant due to the low patient number. We did not observe significant differences in antioxidant genes between IPF patients and controls, although the oxidant-sensitive transcription factor NRF2 (*p* = 0.19), as well as CAT (*p* = 0.23) and SOD2 (*p* = 0.21), appeared to be slightly downregulated in IPF patients, whereas HO-1 and SOD1 tended to be slightly upregulated (Figure 3C).

To assess the effects of putative IPF drugs, cells were treated for 24 h at dose ranges similar to those used in previous studies [13,17,33,34]. Initially, the effects of different dose ranges of pirfenidone (1 mM–100 µM), nintedanib (1–0.01 µM) and saracatinib (10–0.1 µM) on cell viability were analyzed using a MTT cytotoxicity assay to determine mitochondrial metabolic activity as a measure of cell viability. Since the highest concentration of both nintedanib and saracatinib reduced the cell viability below 90% (Figure S1A), these doses were excluded from further study. Next, cells were treated with the highest non-cytotoxic concentrations of the drugs (i.e., 1 mM pirfenidone, 0.1 µM nintedanib and 1 µM saracatinib) and LDH release, an indication of cell damage, was measured. No significant LDH release was determined after treatment for 24 h (Figure S1B), indicating that the concentrations applied were well tolerated for the chosen treatment period.

**Figure 4:**
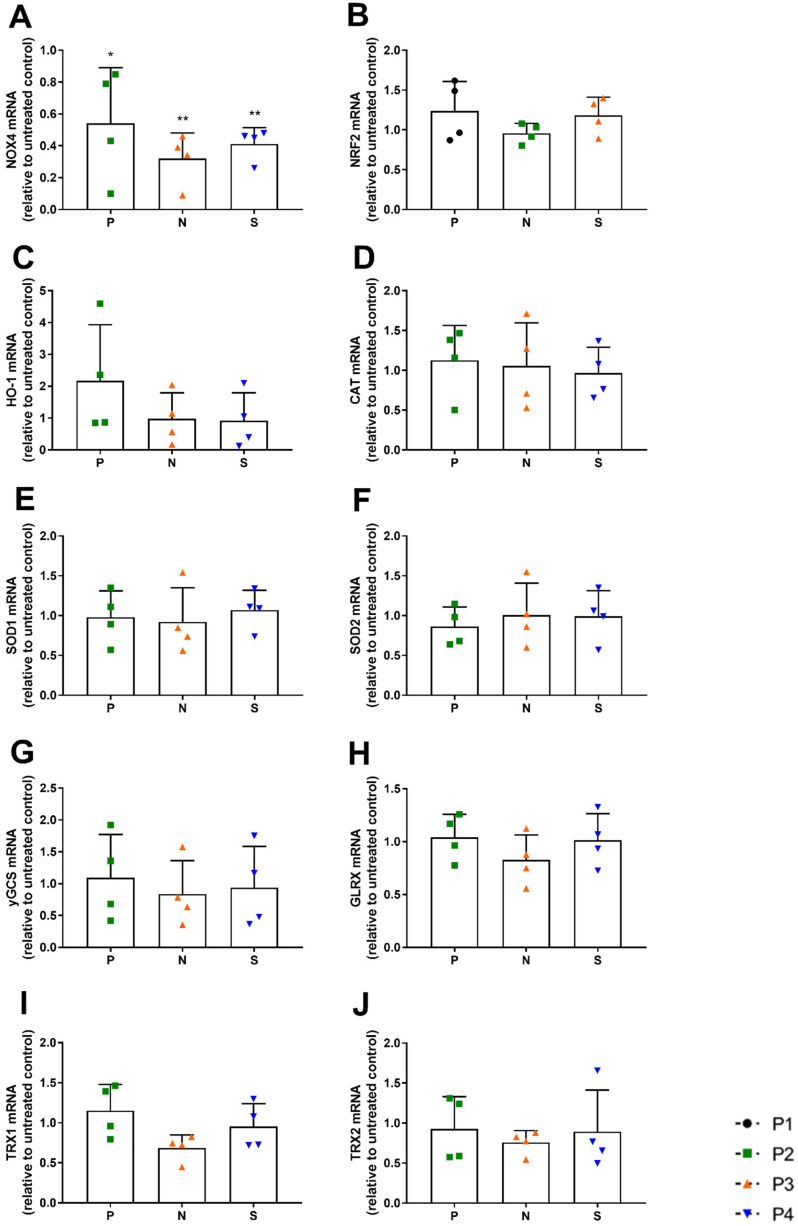
Effect of pirfenidone (P), nintedanib (N) or saracatinib (S) on mRNA expression of NOX4 and antioxidant genes. (**A**) NOX4 expression, (**B**) NRF2 expression, (**C**) HO-1 expression, (**D**) γGCS expression, (**E**) CAT expression, (**F**) SOD1, (**G**) SOD2 expression, (**H**) GLRX expression, (**I**) TRX1 expression and (**J**) TRX2 expression in HBE cells from IPF patients (*n* = 4) after stimulation with pirfenidone (1 mM), nintedanib (0.1 µM) and saracatinib (1 µM) for 24 h. The change in gene expression is expressed as a fold increase compared to the untreated control individually per IPF patient (* *p* < 0.05, ** *p* < 0.01).

Next, we examined the effect of the selected concentrations of pirfenidone, nintedanib and saracatinib on mRNA expression of NOX4 and selected antioxidant genes. All drugs significantly reduced NOX4 expression in IPF patients (Figure 4A), but did not significantly affect NRF2, HO-1, γGCS, SOD1, SOD2, CAT, GLRX, TRX1 and TRX2 expression (Figure 4B–J). Notably, in two patients who had higher expression of NOX4 and lower expression of NRF2, pirfenidone was more effective and did induce the gene expression of various antioxidant genes (Supplemental Table S1).

**Figure 5:**
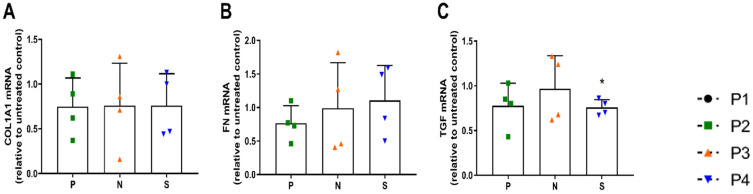
Effect of saracatinib treatment on fibrotic gene expression (**A**) COL1A1 expression, (**B**) FN expression and (**C**) TGF expression in HBE cells from IPF patients (*n* = 4) after stimulation with pirfenidone (P, 1 mM), nintedanib (N, 0.1 µM) and saracatinib (S, 1 µM) for 24 h. The change in gene expression is expressed as a fold increase compared to the untreated control individually per IPF patient (* *p* < 0.05).

IPF development is associated with an increase in fibrotic gene expression, and therefore expression of collagen (COL1A1), fibronectin (FN) and TGF was analyzed. Interestingly, neither pirfenidone nor nintedanib affected COL1A1 (Figure 5A), FN (Figure 5B) or TGF expression, but saracatinib reduced TGF expression significantly (*p* < 0.05, Figure 5C).

**Figure 6:**
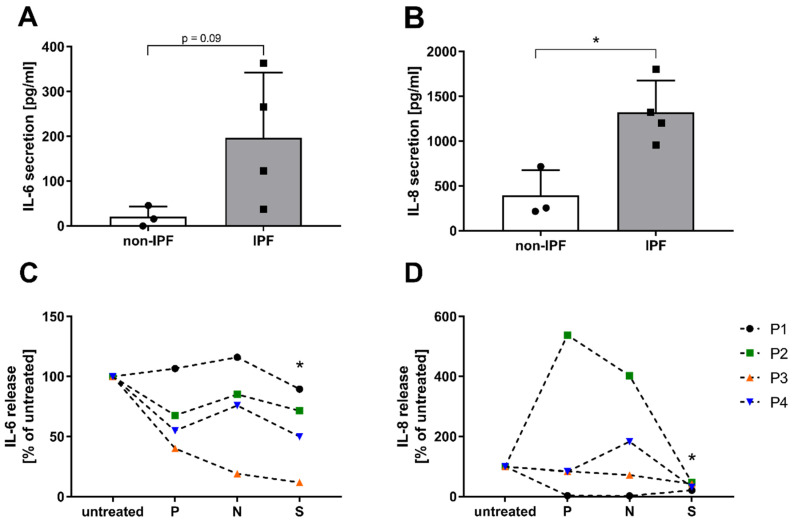
The effect of drug treatment on inflammatory cytokine secretion by HBE cells from patients with IPF (**A**) IL-6 and (**B**) IL-8 secretion in HBE cells from IPF patients (*n* = 4) and controls (*n* = 3), presented as mean ± SD. (* *p* < 0.05). (**C**) IL-6 and (**D**) IL-8 secretion in HBE cells from IPF patients after stimulation with pirfenidone (1 mM), nintedanib (0.1 µM) or saracatinib (1 µM) for 24 h (*n* = 4). Data are presented as a percentage of the untreated cells calculated for each patient (**C**,**D**) (* *p* < 0.05).

As IPF pathology can be accompanied by low-grade chronic inflammation, secretion of pro-inflammatory interleukin (IL)-6 and IL-8 was determined using ELISA. HBE cells isolated from IPF patients did indeed secrete increased levels of IL-6 and IL-8 compared to HBE cells from non-IPF patients (Figure 6A,B). Treatment with pirfenidone and nintedanib had modest effects on HBE cells of IPF patients as IL-6 and IL-8 production was mostly unaffected (Figure 6C,D), but treatment with saracatinib significantly reduced both IL-6 and IL-8 production (*p* = 0.02).

## 4. Discussion

In this pilot study, we demonstrated that ELF-derived cells from patients with IPF and non-IPF controls can be successfully differentiated into a fully stratified bronchial epithelium, consisting of ciliated epithelial, basal, goblet and club cells. Although our previous study revealed differences in basal airway cell expression between patients with IPF compared to non-IPF controls [35], this did not result in major differences in ALI-differentiated cultures of these cells. Our preliminary results indicate increases in NOX4 expression in IPF epithelia that can be reduced by pirfinidone, nintedanib, as well as saracatinib, although their effects on redox homeostasis genes was quite variable between individual patients with IPF. With respect to profibrotic readouts, our data suggest that of the three drugs tested only saracatinib significantly reduced gene expression of the profibrotic growth factor TGF-β. Moreover, we observed that only saracatinib was capable of consistently suppressing inflammatory cytokine release by IPF-derived HBE cells.

IPF is a complex heterogeneous disease, and although there are two drugs licensed, they are not fully effective in every patient, as disease progression still occurs during their therapeutic use [36]. Given the considerable heterogeneity between IPF patients, a more personalized treatment approach may be more suitable and realistic to improve treatment of the affected patients [37]. However, to enable such personalized-medicine approaches, subgroups of IPF patients have to be identified first to help identify the most effective targeted therapies and maximize the outcome of treatment strategies. One possible trait for defining such subgroups is the redox homeostasis, since oxidative stress has been commonly implicated in IPF [38]. Moreover, several genes involved in maintenance of redox homeostasis have shown a diminished expression in patients with IPF [5,28,29,30,31,32]. However, considerable variability in expression of NOX4 and in antioxidant gene expression, as observed in the present study, might explain why redox-targeted therapies have failed to be generally effective in IPF thus far, and may only be suitable for specific clusters of IPF patients instead [39]. Patients with increased NOX4 expression might benefit from combined antioxidant treatment with either of the two antifibrotic drugs pirfenidone or nintedanib, whereas patients without an altered redox balance might rather benefit from a different co-treatment approach. Such an approach fits nicely with the results of a recent meta-analysis assessing the effectiveness of both drugs and the most commonly used antioxidant N-acetyl-cysteine (NAC) in IPF, which showed that the latter may have a role in the treatment of specific clusters of IPF patients only [39]. Alternatively, IPF patients could be genotyped based on the two main subtypes observed in the clinic, i.e., profibrotic and pro-inflammatory, as our results underline that, in some but not all patients, significantly elevated levels of pro-inflammatory cytokines are present that only respond well to saracatinib. Although the role of inflammation within IPF progression is still controversial, partly due to negative multicenter trials of anti-inflammatory drugs within IPF, both the innate and adaptive immune responses are involved and modulated by current antifibrotic drugs [40]. Consequently, it can be anticipated that immunomodulating therapies may be helpful as co-treatment in a special subgroup of IPF patients with increased pulmonary pro-inflammatory cytokine production.

Our pilot findings with respect to the effectiveness of saracatinib in IPF are in line with the recent study of Ahangari et al. in which this SFK inhibitor was shown to revert various fibrogenic pathways, including immune responses and extracellular matrix organization, in both animal and human models of pulmonary fibrosis [17]. Additionally, our previous work has already revealed increased SFK activity mediated through NOX4-dependent oxidation in IPF, leading to enhanced mtROS production and DNA damage [23]. Since SFK are also strongly associated with redox homeostasis, their inhibition could offer a useful new treatment strategy within IPF, possibly in combination with antioxidants to tackle the direct redox-dependent activation of SFK enzymes [23].

Our study is the first study that uses pulmonary bronchial epithelial cells obtained from newly diagnosed and untreated IPF patients to evaluate treatment effects on markers of antioxidant genes and pro-inflammatory cytokine release. Although the current consensus is that alveolar epithelial cells are the key cells driving IPF pathogenesis, there is emerging new evidence that bronchial basal cells are also involved in the development of pulmonary fibrosis. The recent PROFILE study has shown pronounced staining in fibrotic lesions throughout the metaplastic epithelium and enhanced serum levels of three serum proteins, i.e., surfactant protein D, CA19-9 and CA-125 [41]. This finding suggests that these biomarkers reflect an epithelial signature in progressive IPF that is, at least partially, bronchial-driven [41]. This observation fits well with the recent finding that mortality in IPF can be predicted by a nine-gene signature derived from bronchoalveolar lavage (BAL) transcriptome significantly enriched for genes expressed in airway basal cells [42]. Moreover, single-cell RNA sequencing has recently revealed a novel population of IPF-enriched aberrant airway basal-like cells located at the edge of myofibroblast foci and co-express basal epithelial markers, mesenchymal and senescence markers [43]. Finally, one of the most common genetic risk factors yet identified for pulmonary fibrosing diseases is a polymorphism in mucin gene MUC5B that is expressed in the small airway epithelium [44].

Our research, being a pilot study, obviously has several limitations. First, the small sample size is likely to have contributed to our inability to detect other potentially biologically relevant differences, and extended studies with larger patient groups would be needed to fully address this. Second, our IPF patients also differed from our control subjects with respect to age and smoking history, which clearly could confound analysis of lung function and redox homeostasis and potentially influenced the results obtained. Additionally, it would be of interest to also measure ROS levels as well as reactive nitrogen species (RNS) levels in the cultures to directly assess the oxidant burden. Lastly, the working mechanisms of pirfenidone and nintedanib in epithelial cells remain to be elucidated to fully understand and clinically translate our findings. The fact that nintedanib also inhibits non-receptor tyrosine kinases including SFK [45] suggests that part of the working mechanism of this antifibrotic drug might include SFK inhibition. Interestingly, recent observations that saracatinib attenuates profibrotic changes in vitro in human fibroblasts and lung organoids as well as in vivo in mice [17,18], and may actually be more efficacious than pirfenidone or nintedanib, further warrant its possible therapeutic effectiveness for IPF, which is also supported by our present study.

While designing personalized co-treatment strategies for pulmonary fibrosis, the optimal drug-delivery route should be taken into account. Currently, both pirfenidone and nintedanib are orally administered and associated with various unintended side effects. Direct targeted delivery to the lungs might not only reduce these side effects but might also result in higher local drug concentrations, thereby potentially increasing their efficacy [46]. Interestingly, it has recently been shown that lung-targeted delivery of the Nrf2 activator dimethyl fumarate, but not its systemic delivery, boosts antioxidant activity and promotes resolution of age-dependent established lung fibrosis [46]. Future studies with respect to new IPF (co-)therapies including antioxidants should, therefore, also consider lung-targeted drug delivery, as is currently under investigation for aerosolized pirfenidone [47].

**Figure 7:**
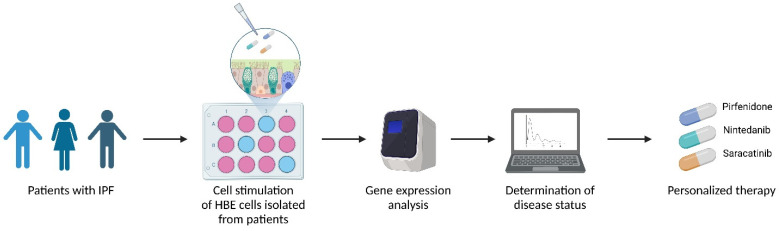
Gene expression analysis based on in vitro treatment of patient-derived bronchial epithelial cells with potential fibrotic drugs may determine disease status, and thus predict possible response towards drugs, before treatment. Created using BioRender.com.

In conclusion, there are still many open questions regarding IPF treatment strategies. This pilot study suggests that treatment responsiveness may vary substantially between individual IPF patients, which may partially be due to a differently affected redox status, and further argues that treatment should be personalized to maximize its health benefits (Figure 7).

## Figures and Tables

**Table 1:** Patient characteristics. Age is expressed in years, DLCO (diffuse capacity of the lung for carbon monoxide), FEV_1_ (forced expiratory volume in 1 s) and FVC (forced vital capacity, also in total volume) in percentage of the predicted value based on age and gender. Data are expressed as a range (mean ± SD). ILD = interstitial lung disease; UIP = usual interstitial pneumonia; NSIP = nonspecific interstitial pneumonia; LUSC = lung squamous cell carcinoma.

ID	Diagnosis	Gender	Age (Years)	DLCO (% Predicted)	FVC (l)	FVC (% Predicted)	Smoking Status
1	ILD, UIP pattern	Male	53	38.1	2.95	78.8	Current, 55 py
2	ILD, UIP pattern	Male	70	36.5	2.39	89.7	Ex-smoker, 50 py
3	ILD, IgG4 associated, NSIP pattern	Male	68	48.4	3.78	60.9	Non-smoker
4	ILD, UIP pattern	Male	77	39.3	3.44	89.4	Ex-smoker, 25 py
5	LUSC	Male	53	101.8	4.08	93	Current, 20 py
6	Lipoma	Female	55	98.4	3.49	113.3	Ex-smoker, 15 py
7	LUSC	Male	55	81.9	4.79	112.7	Current, 25 py

**Table 2:** Human RT-PCR forward and reverse primer sequences.

Gene of Interest	Accession Number	Forward Primer	Reverse Primer
***Actin***	NM_001101.5	CCTGGCACCCAGCACAAT	GCCGATCCACACGGAGTACT
***NOX4***	NM_016931.5	TGGCAAGAGAACAGACCTGA	TGGGTCCACAACAGAAAACA
***NRF2***	NM_006164.5	ACACGGTCCACAGCTCATC	TCTTGCCTCCAAAGTATGTCAA
***HO-1***	NM_002133.3	CTTCTTCACCTTCCCCAACA	GCTCTGGTCCTTGGTGTCAT
***γGCS***	NM_001498.4	CGACCAATGGAGGTGCAGTTA	ACCCTAGTGAGCAGTACCACGAA
***CAT***	NM_001752.4	GATGTGCATGCAGGACAATCAG	GCTTCTCAGCATTGTACTTGTCC
***SOD1***	NM_000454.5	CCACACCTTCACTGGTCCAT	CTAGCGAGTTATGGCGACG
***SOD2***	NM_000636.4	TGGACAAACCTCAGCCCTAACG	TGATGGCTTCCAGCAACTCCC
***GLRX***	NM_002064.3	CACTGCATCCGCCTATACAA	CAGCCACCAACCACACTAAC
***TRX1***	NM_003329.4	GCACGCCAACATTCCAGTTT	ACGCAGATGGCAACTGGTTA
***TRX2***	NM_012473.4	TGGTGGCCTGACTGTAACAC	ACTCAATGGCGAGGTCTGTG
***COL1A1***	NM_000088.4	GGACACAGAGGTTTCAGTGG	CCAGTAGCACCATCATTTCC
***FN1***	NM_212482.4	AGTGGGAGACCTCGAGAAGA	ACTGTGACAGCAGGAGCATC
***TGF-β***	NM_000660.7	CCCTGGACACCAACTATTGC	CTTCCAGCCGAGGTCCTT

## Data Availability

All the data are contained within the article.

## Supplementary Materials

The following supporting information can be downloaded at: https://www.mdpi.com/article/10.3390/antiox12020443/s1.
