# Supplementary material for: Differences in Treatment Response in Bronchial Epithelial Cells from Idiopathic Pulmonary Fibrosis (IPF) Patients: A First Step towards Personalized Medicine?
Source: Antioxidants (Basel). 2023 Feb 10;12(2):443. doi: 10.3390/antiox12020443 (PMC9952618; doi:10.3390/antiox12020443)
Supplement: Supplementary file 1 [file antioxidants-12-00443-s001.zip › antioxidants-2075113-supplementary.pdf]

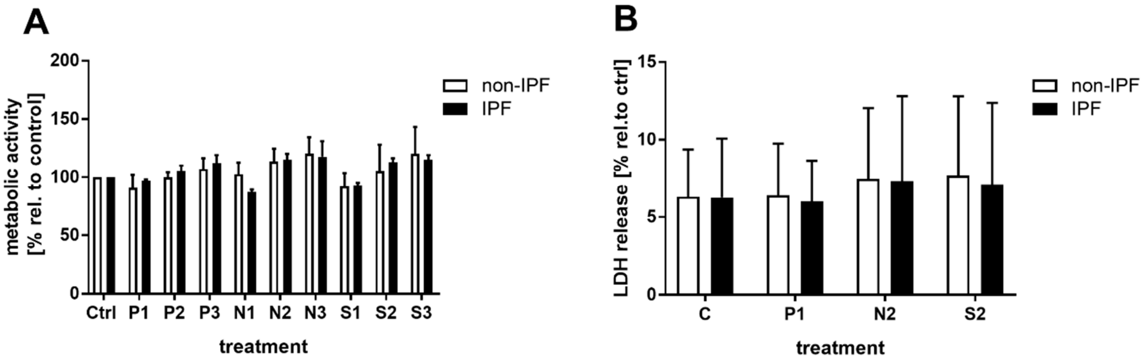

Figure S1. Pirfenidone, nintedanib and saracatinib do not influence cell viability. **(A)** MTT assay and **(B)** LDH assay after treatment with different doses of pirfenidone (P1: 1 mM, P2: 500 µM, P3: 100 µM), nintedanib (N1:1 µM, N2: 0.1 µM, N3: 0.01 µM) and saracatinib (S1: 10 µM, S2: 1 µM, S3: 0.1 µM) for 24 hrs in IPF patients (n = 4) and controls (n = 3). Data are presented as mean ± SD.

Table S1. Gene expression of NOX4, NRF2, HO-1, γGCS, SOD1, SOD2, CAT, GLRX, TRX1 and TRX2 for individual IPF patients without and with treatment with pirfenidone (1mM), nintedanib (0.1 µM) and saracatinib (1 µM) for 24 hrs. Fold increase was expressed compared to untreated non-IPF controls.

	NOX4	NRF2	HO-1	γGCS	SOD1	SOD2	CAT	GLRX	TRX1	TRX2
**Patient 1**	8,59	0,43	0,95	0,24	0,44	3,11	0,36	1,04	0,29	0,37
**P**	6,77	0,64	2,25	0,32	0,49	3,06	0,53	1,31	0,41	0,49
**N**	3,93	0,39	1,09	0,19	0,33	2,69	0,26	0,78	0,21	0,31
**S**	4,16	0,60	2,00	0,42	0,59	4,20	0,50	1,39	0,38	0,62
**Patient 2**	2,24	0,93	2,22	2,07	1,07	1,18	1,21	1,18	0,94	1,05
**P**	1,91	0,81	1,89	0,87	0,61	0,81	0,61	1,38	0,75	0,62
**N**	0,89	0,75	1,26	0,74	0,60	1,20	0,64	1,32	0,42	0,57
**S**	0,58	0,83	0,27	0,76	0,79	1,25	0,79	1,26	0,68	0,69
**Patient 3**	3,74	0,79	1,24	0,86	0,77	1,34	0,61	1,51	0,75	0,77
**P**	0,38	1,28	5,68	1,66	1,04	0,86	0,84	1,46	1,09	0,96
**N**	0,34	0,82	2,53	1,36	1,18	0,80	1,04	1,33	0,62	0,68
**S**	1,71	0,88	1,30	1,00	0,86	0,76	0,46	1,10	0,54	0,59
**Patient 4**	1,37	0,38	3,54	1,22	0,33	1,18	0,18	4,86	0,77	4,59
**P**	0,59	0,36	3,08	0,83	0,29	1,35	0,21	3,76	0,74	2,64
**N**	0,47	0,41	0,62	0,77	0,28	1,83	0,23	2,71	0,57	3,55
**S**	0,62	0,50	1,41	0,58	0,36	1,17	0,19	4,54	0,83	2,28
